# One-Step and Colorimetric Detection of Fish Freshness Indicator Hypoxanthine Based on the Peroxidase Activity of Xanthine Oxidase Grade I Ammonium Sulfate Suspension

**DOI:** 10.3389/fmicb.2021.791227

**Published:** 2021-12-02

**Authors:** Chen Guo, Shuhan You, Changmei Li, Tiantian Chen, Xiudan Wang

**Affiliations:** ^1^Shandong Provincial Key Laboratory of Biochemical Engineering, College of Marine Science and Biological Engineering, Qingdao University of Science and Technology, Qingdao, China; ^2^CAS Key Laboratory of Marine Ecology and Environmental Sciences, Institute of Oceanology, Chinese Academy of Sciences, Qingdao, China

**Keywords:** hypoxanthine, fish freshness, colorimetric, one-step detection, XOD-ASS

## Abstract

The global food waste problem, especially aquatic product spoilage, stimulates the accurate freshness analysis of food products. However, it still remains a great challenge to realize in-field determination of fish freshness at the time of use. In the present study, a colorimetric enzyme biosensor was developed for one-step detection of hypoxanthine (Hx), which is an important intermediate of adenosine triphosphate decomposition during fish storage. We demonstrate that xanthine oxidase grade I ammonium sulfate suspension (XOD-ASS) possesses peroxidase activity. It can oxidize different peroxidase substrates, including 3,3′,5,5′-tetramethylbenzidine, 2,2′-azino-bis(3-ethylbenzothiazoline-6-sulfonic acid) diammonium salt, and o-phenylenediamine in the presence of H_2_O_2_, producing visible color reactions. Further experiments indicate that XOD-ASS displayed effective peroxidase activity and could be used for H_2_O_2_ detection. Based on this, a one-step Hx detection method was established using only XOD-ASS as the catalyst. The method displays a good linear relationship in the range from 20 to 100 μM with a detection limit of 6.93 μM. Additionally, we successfully applied this method in testing Hx accumulation in sea bass fish samples of different storage times. The recovery values range from 97.44 to 102.56%. It is exciting to note that, compared with other methods, our proposed method provides a robust advantage on the economic reaction system, ease of preparation, short time consumption, and moderate reaction temperature. We believe that this method shows good application prospects for on-site fish freshness determination.

## Introduction

Nowadays, there are increasing requirements and regulations in the fields of biotechnology control, environmental protection, and food/water quality certification (Flachsbarth et al., 2015). A rising number of clinical diagnoses and veterinary tests are required regarding human and animal health (Görgülü et al., 2013). Therefore, developing rapid, easy, economic, and accurate analysis methods may aid in these processes and general laboratory tasks.

In the food industry, especially aquatic products, which are highly perishable with a limited shelf-life after slaughter, the global waste problem stimulates the accurate freshness analysis of food products (Prabhakar et al., 2020). Traditionally, to meet the freshness standard, consumers mainly rely on a sensory approach to discriminate fresh fish products, and it is highly dependent on consumers’ level of perception and experience. Fishing factories try to record the capture or slaughter date of the products, but different storage or processing methods can greatly affect fish freshness. These methods are rapid and easy to carry out, but they are unreliable to assess fish freshness especially before the initial stages of spoilage.

Recently, estimating fish freshness has been widely investigated at the chemical, biochemical, and microbiological levels (Gil et al., 2011; Prabhakar et al., 2020). Various biomarkers, such as trimethylamine (TMA) and total volatile basic nitrogen (TVB-N), metabolites of adenosine triphosphate (ATP) degradation, and microbial count and activity were established as indicators of fish freshness (Cela-Pérez et al., 2015; Cheng and Sun, 2015; Peleg, 2016). TMA and TVB-N are widely preferred for assessment of fish quality and shelf life, but they lag behind as great indicators because they mainly indicate the later stages of fish spoilage (Sykes et al., 2009). Nucleotide and nucleoside metabolites produced by ATP decomposition has been demonstrated as one of the most important reasons that affect the freshness and quality of fish products (Shen et al., 1996; Carsol et al., 1997; Albelda et al., 2017). Compared with other indicators, hypoxanthine (Hx), which is an important intermediate of ATP metabolism, accumulates right after the fish is slaughtered (Cela-Pérez et al., 2015; Albelda et al., 2017). Therefore, the evaluation of Hx has the potential to determine the early stages of fish spoilage.

Hypoxanthine determination can be achieved using classic methods, such as high performance liquid chromatography (Fukuuchi et al., 2013; Qu et al., 2017) and spectrophotometric measurements (Amigo et al., 2005; Weeranantanaphan et al., 2011). These methods, in general, require expensive equipment and skilled technicians, which confines them to specialized laboratories. The emerging use of biosensors has the potential to provide simple and rapid determination platforms to overcome these challenges. The majority of developed biosensors involve a reaction that xanthine oxidase (XOD) could catalyze the oxidization of Hx and xanthine in the presence of oxygen to generate hydrogen peroxide (H_2_O_2_) and uric acid (UA) (Kelley et al., 2010). Then, the Hx or xanthine concentration can be determined by measuring the produced H_2_O_2_, UA or consumed O_2_, producing an electrochemical, fluorescent, or colorimetric signal (Görgülü et al., 2013; Wu et al., 2018; Chen et al., 2020). Electrochemical biosensors can achieve fast and easy detection, but they require a potentiostat to measure the voltammetric current (Görgülü et al., 2013; Albelda et al., 2017). Fluorescent and colorimetric sensors have been widely developed by combining XOD with peroxidase or nanoenzymes with peroxidase-like activity. For example, Li et al. (2008) develop a spectrophotometric quantitation method to detect xanthine by conjugating XOD with horseradish peroxidase. A series of colorimetric or fluorescent biosensors based on the production of H_2_O_2_ by XOD and peroxidase-like catalytic activity of nanoenzymes, including platinum nanoparticles (Chen et al., 2020), selenium-doped graphite carbon nitride (Qiao et al., 2015), and amino-functionalized metal organic framework (Hu et al., 2018) are also established, contributing to the rapid freshness evaluation of aquatic products. In these reactions, a two-step catalytic reaction is adopted: First, XOD catalyzes the reaction of Hx or xanthine with oxygen, yielding H_2_O_2_; second, H_2_O_2_ participates in the peroxide reaction catalyzed by a peroxidase or peroxidase mimic enzyme.

In the present study, we find that XOD grade I ammonium sulfate suspension (XOD-ASS) possesses peroxidase activity; it can not only catalyze the oxidization of Hx or xanthine to produce H_2_O_2_, but catalyze the oxidization of 3,3′,5,5′-tetramethylbenzidine (TMB) (colorless) by H_2_O_2_ to produce oxidized TMB (blue). Based on this, we further propose an easy, one-step, colorimetric method for Hx detection using only XOD-ASS as the catalyst. We demonstrate the utility of our method in fish samples and realize on-site, quantitative detection of Hx, providing a cheap, easy fish-freshness evaluation method.

## Materials and Methods

### Materials

Xanthine oxidase grade I ammonium sulfate suspension was purchased from Sigma-Aldrich (St. Louis, MO, United States) and used as received. Hx standard of chromatographically pure was obtained from Solarbio (Beijing, China). UA, 30% H_2_O_2_, glucose, glycine, ascorbic acid, cysteine, and inosine were provided by Aladdin Reagents (Shanghai, China). TMB, 2,2′-azino-bis(3-ethylbenzothiazoline-6-sulfonic acid) diammonium salt (ABTS), and o-phenylenediamine (OPD) were bought from Macklin Biochemical (Shanghai, China). Other chemicals were of analytical grade, and all compounds used in this work were prepared without any further purification. All solutions were prepared with distilled deionized water purified by a Milli-Q Purification System (Millipore, MA, United States).

### The Oxidation of 3,3′,5,5′-Tetramethylbenzidine Catalyzed by Xanthine Oxidase Grade I Ammonium Sulfate Suspension

3,3′,5,5′-tetramethylbenzidine and H_2_O_2_ were used to examine the peroxidase activity of XOD-ASS. The reaction was performed in a 100-μL system (20 mM sodium citric buffer, pH = 5.0) containing 0.1 U/mL XOD-ASS (one unit converts 1.0 μmole of xanthine to UA per min at pH 7.5 at 25°C), 1 mM TMB, and 1 mM H_2_O_2_ and incubated at room temperature for 10 min. Then, the UV-vis spectrum of 400 to 800 nm was recorded with a microplate reader (Tecan i-control, Infinite M1000 PRO, Switzerland). The steady-state kinetics of XOD was recorded at 652 nm using a time-scan model of the microplate reader. Reactions without XOD-ASS or H_2_O_2_ were set as controls. The peroxidase activity of XOD-ASS was further verified using two other peroxidase substrates, OPD and ABTS, and the reaction system used for OPD and ABTS was the same as that for TMB. To obtain the optimal reaction conditions, the reaction was first incubated at a temperature from 20°C to 65°C and then in different buffer solutions with pH values from 3 to 10. Standard reaction conditions were then adopted with varying concentrations of TMB (0.1-2 mM) at a fixed concentration of H_2_O_2_ (1 mM) or varying concentrations of H_2_O_2_ (0.2-10 mM) at a fixed concentration of TMB (1 mM).

### Hypoxanthine Detection Using Xanthine Oxidase Grade I Ammonium Sulfate Suspension and Selectivity Study

For Hx detection, the concentration of XOD-ASS was optimized from 0.0125 to 0.2 U/mL (concentrations of XOD), and the concentration of TMB was optimized from 0.0125 to 1.0 mM. The reaction temperature was optimized at 25°C, 37°C, and 50°C. At the optimal reaction condition, Hx detection was performed in a 100-μL system (20 mM sodium citric buffer, pH = 5.0) with variable concentrations of Hx from 0.02 to 1.0 mM. After incubation at room temperature for 10 min, the absorbance signal at 652 nm was recorded by the microplate reader.

To determine the selectivity of the detection method, a series of common substrates, including glucose, glycine, ascorbic acid, UA, cystine, and inosine with concentrations of 1 mM were adopted as interferences.

### Detection of Hypoxanthine in Fish Samples

Live sea bass was bought from the local market and slaughtered to obtain fresh fish filets. In total, 24 pieces of fish filet (about 10 g/each) were randomly divided into eight groups and were left to decay for 0, 1, 3, 6, 12, 24, 36, and 48 h at room temperature, respectively. At each time point, three replicates of fish filet were, respectively, minced with a mortar and pestle and stored at −20°C until analysis. For analysis, a fish meat sample of 1 g was mixed with 10 mL distilled water and homogenized using an ultrasonic homogenizer for 15 min. The homogenous mixture was centrifuged at 12,000 rpm for 10 min at 4°C, and the obtained supernatant was diluted three times with distilled water. Two samples of fish extract (3 and 6 h) were spiked with standard Hx solutions with final concentration of 10, 15, and 25 μM. To perform Hx analysis, 20 μL of the fish extract (or spiked fish extract) was mixed with 80 μL sodium citric buffer system (the final mixture contained 0.025 U/mL XOD-ASS, 0.2 mM TMB, and 20 mM sodium citric). The mixture was incubated at room temperature for 10 min, and the absorbance signal at 652 nm was recorded by the microplate reader.

### Statistical Analysis

All experiments were performed at least in triplicate, and the results were presented as mean ± standard deviation and analyzed by SPSS 16.0. Significant differences among groups were evaluated by one-way analysis of variance (ANOVA) and linear regression analysis was performed. It was considered significantly different at *p* < 0.05. Origin Pro 7.5 software was used to prepare the figures.

## Results and Discussion

### Xanthine Oxidase Grade I Ammonium Sulfate Suspension Catalyzes the Oxidation of Peroxidase Substrates

**FIGURE 1:**
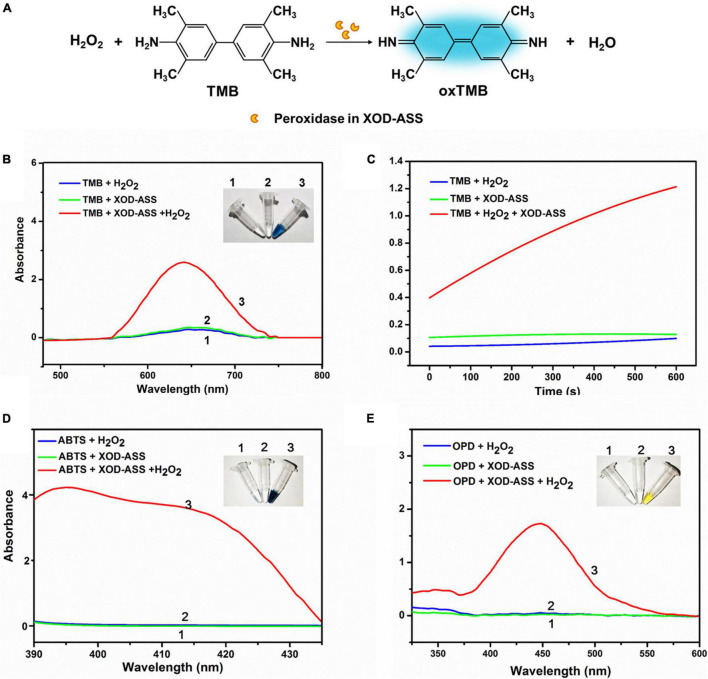
XOD-ASS shows peroxidase activity. **(A)** Scheme illustration of oxidation of TMB into oxidized TMB by XOD-ASS. **(B)** The absorbance spectrum of XOD-ASS catalyzing the oxidation of TMB in the presence of H_2_O_2_. Inset represents the corresponding colorimetric result of the reactions. **(C)** The time-dependent absorbance curve at 652 nm of the reactions. **(D)** The absorbance spectrum of XOD-ASS catalyzing the oxidation of ABTS in the presence of H_2_O_2_. Inset represents the corresponding colorimetric result of the reactions. **(E)** The absorbance spectrum of XOD-ASS catalyzing the oxidation of OPD in the presence of H_2_O_2_. Inset represents the corresponding colorimetric result of the reactions.

Typically, peroxidase catalyzes the oxidation of TMB and displays a significant color change from colorless to blue in the presence of H_2_O_2_ (Gao et al., 2007). First, we investigated the peroxidase activity of XOD-ASS using the typical peroxidase substrate TMB (Figure 1A). The result demonstrates that XOD-ASS catalyzed the reaction of substrate TMB in the presence of H_2_O_2_, producing oxidized TMB with a blue color (Figure 1B, inset). No color signal was observed for the reactions without XOD-ASS or H_2_O_2_, indicating XOD-ASS and H_2_O_2_ were both necessary for the oxidation of TMB. Spectroscopic analysis revealed the maximum absorption wavelength at 652 nm (Figure 1B), which corresponded with previous research (Gao et al., 2007; Yang et al., 2019). At the same time, the time course curves of the reactions were monitored at 652 nm (Figure 1C) within 600 s, confirming that the catalysis of the reaction was time-dependent, and a minimal signal was observed in the absence of XOD-ASS or H_2_O_2_. The peroxidase activity of XOD-ASS was further characterized by replacing TMB with other substrates ABTS and OPD. Figures 1D,E shows that XOD-ASS could not only catalyze the oxidation of TMB, but also ABTS to produce a color change from colorless to dark green and OPD to produce a yellow color. These results confirm that XOD-ASS possesses peroxidase activity toward three different peroxidase substrates.

Based on the spectroscopic and colorimetric results, we deduce that XOD-ASS might contain lactoperoxidase (LPO) from milk. To verify our deduction, sodium dodecyl sulfate-polyacrylamide gel electrophoresis (SDS-PAGE) was performed to detect the protein components of XOD-ASS. As shown in Supplementary Figure 1, multiple bands were identified in the SDS-PAGE result, demonstrating that XOD-ASS is a crude extract of bovine milk. Particularly, the two bands with the largest quantity are about 150 and 75 kDa, corresponding to the molecular weight of XOD (Enroth et al., 2000) and LPO (Atasever et al., 2013), respectively. LPO is the second most abundant enzyme in bovine milk after XOD, and the concentration can be as high as 1–19 U/mL milk (de Wit and van Hooydonk, 1996). They act together *in vivo* to generate reactive oxygen species and reactive nitrogen species, which is referred to as the “XOD-LPO” system (Al-Shehri et al., 2020). Therefore, it is reasonable that XOD-ASS contains LPO. Considering the lower cost and easier preservation, XOD-ASS might have the potential to develop a one-step Hx detection method using only XOD-ASS as the catalyst without conjugating XOD with other peroxidases or peroxidase mimic enzymes.

### Steady-State Kinetics Assay

**FIGURE 2:**
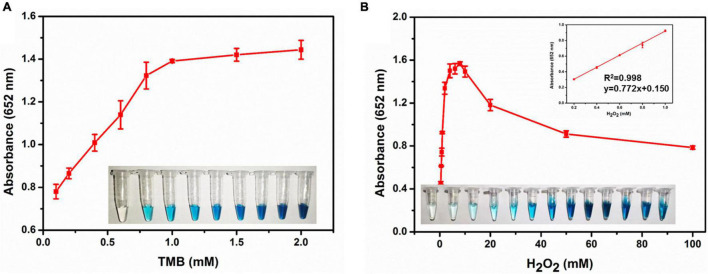
**(A)** The absorbance at 652 nm in different concentrations of TMB. Inset represents the corresponding colorimetric result of the reactions. **(B)** The absorbance at 652 nm in different concentrations of H_2_O_2_. Inset represents the corresponding colorimetric result of the reactions and the linear calibration plot for H_2_O_2_.

On the basis of the peroxidase catalytic activity of XOD-ASS, we investigated the optimal reaction condition of the oxidation of TMB catalyzed by XOD-ASS. As shown in Supplementary Figure 2, the relative activity reached the maximum at 50°C and pH 5.0, respectively. Then, substrate-dependent kinetic analysis was carried out by changing the concentration of one substrate while the concentration of another one was kept constant. As shown in Figure 2A, the absorbance value at 652 nm increased with substrate TMB ranging from 0.1 to 1 mM, and the value came to a plateau at higher concentrations. The color variation for the TMB response could be seen with the naked eye (Figure 2A inset). The color and the absorbance value were positively correlated with substrate H_2_O_2_ ranging from 0.2 to 8 mM, but a higher concentration could inhibit the oxidation reaction (Figure 2B). The absorbance value at 652 nm was linear with H_2_O_2_ concentration in the range from 0.2 to 1.0 mM with a correlation coefficient (*R*^2^) of 0.998. The linear equation was calculated to be *y* = 0.772x + 0.150 (*y* represents absorbance 652 nm, and x represents the concentration of H_2_O_2_). Our results demonstrate that XOD-ASS contains effective peroxidase activity and could be used for H_2_O_2_ detection.

### Sensitivity and Specificity for Detection Hypoxanthine

**FIGURE 3:**
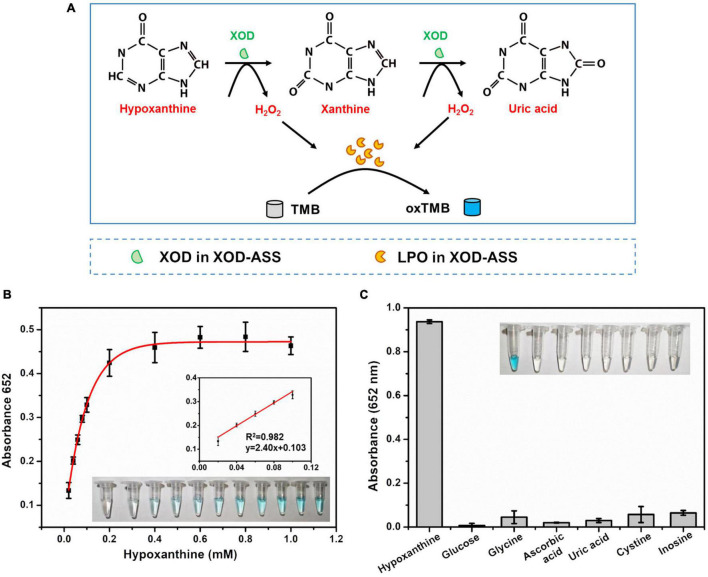
**(A)** Schematic illustration of one-step and colorimetric detection of Hx catalyzed by XOD-ASS. **(B)** The absorbance at 652 nm in different concentrations of Hx. Inset represents the corresponding colorimetric result of the reactions and the linear calibration plot for Hx. **(C)** Selectivity of the established one-step Hx detection method.

We further develop a one-step Hx detection method using the XOD-ASS mixture as the catalyst. XOD-ASS could not only catalyze the oxidization of Hx or xanthine to produce H_2_O_2_ (XOD functions in this reaction), but catalyzes the oxidization of TMB (colorless) by H_2_O_2_ to produce oxidized TMB (blue) (LPO might function in this reaction) (Figure 3A). The parameters that might affect the performance of the detection were optimized (Supplementary Figure 3), and the optimal conditions were adopted to detect Hx. As shown in Figure 3B, the color variation of TMB oxidation was dependent on the concentration of Hx, and the absorbance at 652 nm was regularly enhanced with increasing Hx concentration. The corresponding calibration curve (Figure 3B inset) displayed the linear relationship between the absorbance value and Hx concentration in the range from 20 to 100 μM (*R*^2^ = 0.998, *p* = 0.00091 < 0.05). The detection limit was calculated based on a triple standard deviation of blank samples (S/N = 3, S represents sensitivity and N represents noise), and it was determined to be 6.93 μM, which is much lower than the threshold of Hx concentration in fresh aquatic products and could satisfy the application in evaluating fish freshness (Chen et al., 2020). According to a previous study, Hx concentration is lower than 529 μM in fresh aquatic products (Chen et al., 2020), so the analytical sensitivity of our method is sufficient for aquatic product freshness evaluation. The selectivity performance of our established one-step Hx detection method was further investigated by determining the effect of potentially coexisting substances, including glucose, glycine, ascorbic acid, UA, cysteine, and inosine (Figure 3C). The results indicate that no absorbance was obtained for these substances except Hx, demonstrating good selectivity of our XOD-ASS–based, one-step Hx detection method.

### Method Validation and Detection of Hypoxanthine in the Fish Products

A standard addition method was further applied to evaluate the effectiveness of our method. Different concentrations of Hx (10, 15, and 25 μM) were added to diluted fish extracts. As shown in Table 1, our results show good recoveries from 97.44% to 102.56%, demonstrating satisfactory applicability of the method in real sample detection.

**TABLE 1:** Analytical results of Hx and spiked Hx in fish extracts.

Sample	Original (μM)	Spiked (μM)	Found (μM)	Recovery (%)	RSD (%)
Sample 1 (3 h)	19.04	10	28.91	98.72	4.44
		15	33.65	97.44	5.13
		20	39.29	101.28	2.94
Sample 2 (6 h)	27.37	10	37.63	102.56	2.22
		15	41.99	97.44	2.56
		20	46.99	98.08	3.33

**FIGURE 4:**
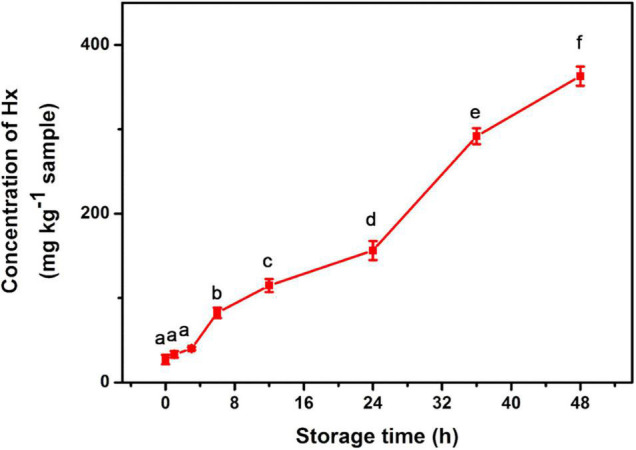
Detection of Hx in sea bass fish sample during storage for 48 h at room temperature. Different lowercase letters indicate Hx concentrations in fish samples of different storage time are significantly different (*p* < 0.05).

To explore the practical application of the developed method, sea bass fish samples at various times ranging from 1 to 48 h after death were tested for Hx accumulation. It is known that Hx starts to accumulate immediately following ATP degradation after the fish is slaughtered (Cela-Pérez et al., 2015). Our assay exhibited a gradually increasing trend during the storage process of the sea bass fish sample (Figure 4). Similar trends of Hx were also observed in fish such as tilapia and shrimp in previous reports (Chen et al., 2020; Mustafa et al., 2021), indicating the high efficiency and potential application of the method in real sample extracts. The obtained concentration of Hx can be related to fish-freshness monitoring. Previous studies give suggested levels of Hx in subfresh and decayed fish. For example, Qiong et al. (1998) suggest that, for *Mylopharyngodon piceus*, an Hx concentration (C_*Hx*_) of ≤72 mg/kg indicates fresh; 72 mg/kg < C_*Hx*_ ≤ 118 mg/kg indicates subfresh; >118 mg/kg indicates spoiled. According to the national standard of China, aquatic products are considered to be fresh when the concentration of Hx is lower than 529 μM (Chen et al., 2020). Based on these suggestions and standards, sea bass fish samples were considered fresh during 0–6 h (C_*Hx*_ lower than 82 mg/kg) storage at room temperature, started to deteriorate during 6–12 h (C_*Hx*_ of 82–115 mg/kg, subfresh, edible), and became spoiled after 12 h (no longer dietary is suggested), but these levels need further validation with TVB-N levels, and they may vary in other types of fish. Therefore, it is essential to establish a database of Hx concentrations for different kinds of fish, and this method will just meet future need as a general platform for freshness screening.

We compare our one-step Hx detection method with previously reported biosensors, which conjugate XOD with peroxidase or peroxidase-mimic nanoenzymes (Table 2). Compared with these methods, our proposed method requires a one-step reaction and 10 min room temperature incubation to realize the colorimetric detection of Hx. Besides this, the method involves only one catalyst XOD-ASS, which is less expensive than other forms of XOD as revealed by the Sigma official website. Based on the price of XOD-ASS and the reagents used, a cost estimate of the XOD-ASS–based solution assay per sample is about ¥0.2 ($0.03), and the cost of nanoenzyme/XOD biosensor per sample is about $0.11 and $3.4 per test for a commercial assay (Mustafa et al., 2021). Therefore, the established one-step Hx detection method provides a robust advantage on economic reaction system, short time consumption, and moderate reaction temperature, providing a simple choice for on-site fish freshness determination.

**TABLE 2:** Comparison of different Hx detection methods.

Catalysts	Steps	Target	Reaction temperature and time	Result readout	LOD	References
XOD + HRP	2	Hx	37°C, 8 min	A508	0.05 mM	Li et al., 2008
XOD + Selenium dopedgraphitic carbon nitride nanosheets	2	Hx	(1) 25°C, 60 min (2) 25°C, immediately	A652	0.016 μM	Qiao et al., 2015
XOD + Amino-functionalized metal organic framework	2	Hx	(1) 25°C, 40 min (2) 25°C, 10 min	Fluorescence intensity	3.93 μM	Hu et al., 2018
XOD + Platinum nanoparticles	2	Hx	(1) 37°C, 30 min (2) 37°C, 30 min	Fluorescence intensity	2.88 μM	Chen et al., 2020
XOD + BSA-stabilized Au clusters	2	Xanthine	(1) 37°C, 15 min (2) 40°C, 10 min	A652	0.5 μM	Wang et al., 2011
XOD + MoSe_2_ nanosheets	2	Xanthine	(1) 25°C, 20 min (2) 25°C, immediately	A652	1.96 μM	Wu et al., 2018
XOD-ASS	1	Hx	25°C, 10 min	A652	6.93 μM	This work

## Conclusion

In this work, we first report that XOD-ASS contains effective peroxidase (LPO) and can be used for H_2_O_2_ detection. Based on this, a simple, inexpensive, and one-step Hx detection method using only XOD-ASS as the catalyst is established. The quantification of Hx displays a linear range of 20–100 μM with a detection limit of 6.93 μM. Besides this, the method has a good selectivity and shows satisfactory applicability in fish sample evaluation with good recoveries. Considering the low cost, ease of preparation, and visual detection, this method shows good application prospects for on-site fish-freshness determination.

## Data Availability Statement

The original contributions presented in the study are included in the article/Supplementary Material, further inquiries can be directed to the corresponding author/s.

## Author Contributions

CG, SY, and CL designed and conducted the experiments. TC and XW conceived and supervised the project. XW managed the funding acquisition. CG and SY compiled and analyzed the output data, and designed and wrote the first version of the manuscript. All authors edited and approved the final version of the manuscript.

## Conflict of Interest

The authors declare that the research was conducted in the absence of any commercial or financial relationships that could be construed as a potential conflict of interest.

## Publisher’s Note

All claims expressed in this article are solely those of the authors and do not necessarily represent those of their affiliated organizations, or those of the publisher, the editors and the reviewers. Any product that may be evaluated in this article, or claim that may be made by its manufacturer, is not guaranteed or endorsed by the publisher.
