# Supplementary material for: One-Step and Colorimetric Detection of Fish Freshness Indicator Hypoxanthine Based on the Peroxidase Activity of Xanthine Oxidase Grade I Ammonium Sulfate Suspension
Source: Front Microbiol. 2021 Dec 2;12:791227. doi: 10.3389/fmicb.2021.791227 (PMC8672161; doi:10.3389/fmicb.2021.791227)
Supplement: Supplementary file 1 [file Data_Sheet_1.docx]

Supplementary Material

1. **Sodium dodecyl sulfate - polyacrylamide gel electrophoresis (SDS-PAGE) of XOD-ASS**

The XOD-ASS was diluted 10 times using water to the final protein concentration about 1.8 mg/mL and discontinuous SDS-PAGE was performed under denatured conditions. The stacking and separating gels contained 50 and 150 g L^−1^ acrylamide (Sangon, Shanghai, China) respectively. An initial voltage of 80 V was applied, which was increased to 120 V when the tracking dye reached the separating gel. Gels were stained by immersion in Coomassie Brilliant Blue R-250 (1 g L^−1^, in a mixture of methanol, acetic acid and water with a volume ratio of 1 : 2 : 17) for 20 min and then immersed in destaining solution (methanol : acetic acid : water=1 : 1 : 8, v : v : v) overnight. Images were captured using an HP Scanjet.

As shown in Fig. S1, multiple bands were identified in the SDS-PAGE result. The two bands with the largest quantity are about 150 kDa and 75 kDa. According to previous report^1^, XOD is a homodimer consisting of two 150 kDa subunits. The predicted molecular weight of bovine LPO is 78 kDa, corresponding to the band about 75 kD^2^.


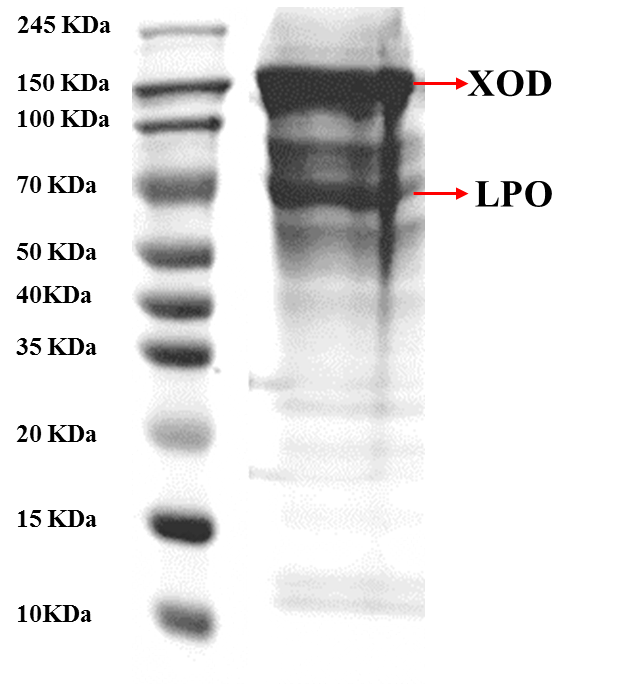


**Supplementary Figure 1.**SDS-PAGE of diluted XOD-ASS.

1. **pH and temperature dependence of the oxidation of TMB catalyzed by XOD-ASS**

Enzymes usually have an optimum pH (or pH range) and temperature at which their catalytic activity is the highest. The optimal reaction condition of the oxidation of TMB catalyzed by XOD-ASS was explored by investigating the relative peroxidase activity of XOD at pH in the range of 3–10 and temperature in the range of 20–65°C. As shown in Fig. S1, the relative activity reached the maximum at 50°C and pH 5.0, respectively.


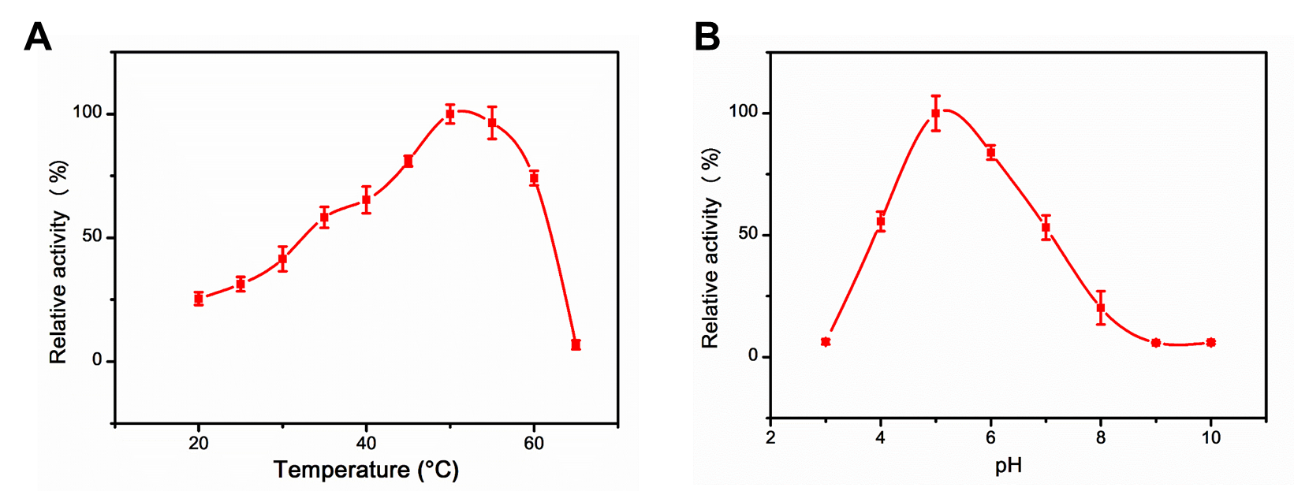


**Supplementary Figure 2.** The peroxidase activity of XOD-ASS is pH and temperature dependent.

1. **Optimization of one-step hypoxanthine detection**

For hypoxanthine detection, the amount of enzyme was optimized from 0.0125U/mL to 0.2 U/mL and the concentration of TMB was optimized from 0.0125 mM to 1.0 mM. The reaction temperature was optimized at 25°C, 37°C and 50°C. As shown in Fig. S2A, the absorbance at 652 nm firstly increased with the dosage of XOD and reached the maximum at 0.025 U/mL. But it displayed a decreasing trend at higher dosage of XOD. So 0.025 U/mL XOD was adopted in the detection process. The effect of the concentration of TMB on the absorbance was also investigated. The absorbance value at 652 nm increased with substrate TMB ranging from 0.05-1.0 mM at a fixed concentration of H_2_O_2_ and the value came to a plateau at higher concentrations. From the point of economy, the final concentration of 0.2 mM was chosen as the optimum concentration of TMB. To determine the temperature, we performed the detection at three temperatures including 25°C, 37°C and 50°C. Among them, 25°C is the room temperature which is easily to realize without any heater, 37°C is the optimum temperature for the XOD to catalyze the oxidation of hypoxanthine and 50°C is the optimum temperature for the XOD to catalyze the oxidation of TMB in the presence of H_2_O_2_. As shown in Fig S2C, when the reaction was performed at 25°C or 37°C the absorbance value at 652 nm is significant higher (*p*< 0.05) than that when the reaction was performed at 50°C. Considering the convenience to operate and energy economy, 25°C was chosen as the optimum temperature to detect hypoxanthine. In the present study, pH value at 5.0 was demonstrated to optimal for the peroxidation activity in this study and previous study (Yang et al., 2019) demonstrated the oxidation of TMB by H_2_O_2_ could be suppressed at lower pH (pH < 3.0) or in alkaline solution with too high pH value. So pH 5.0 was adopted.

**
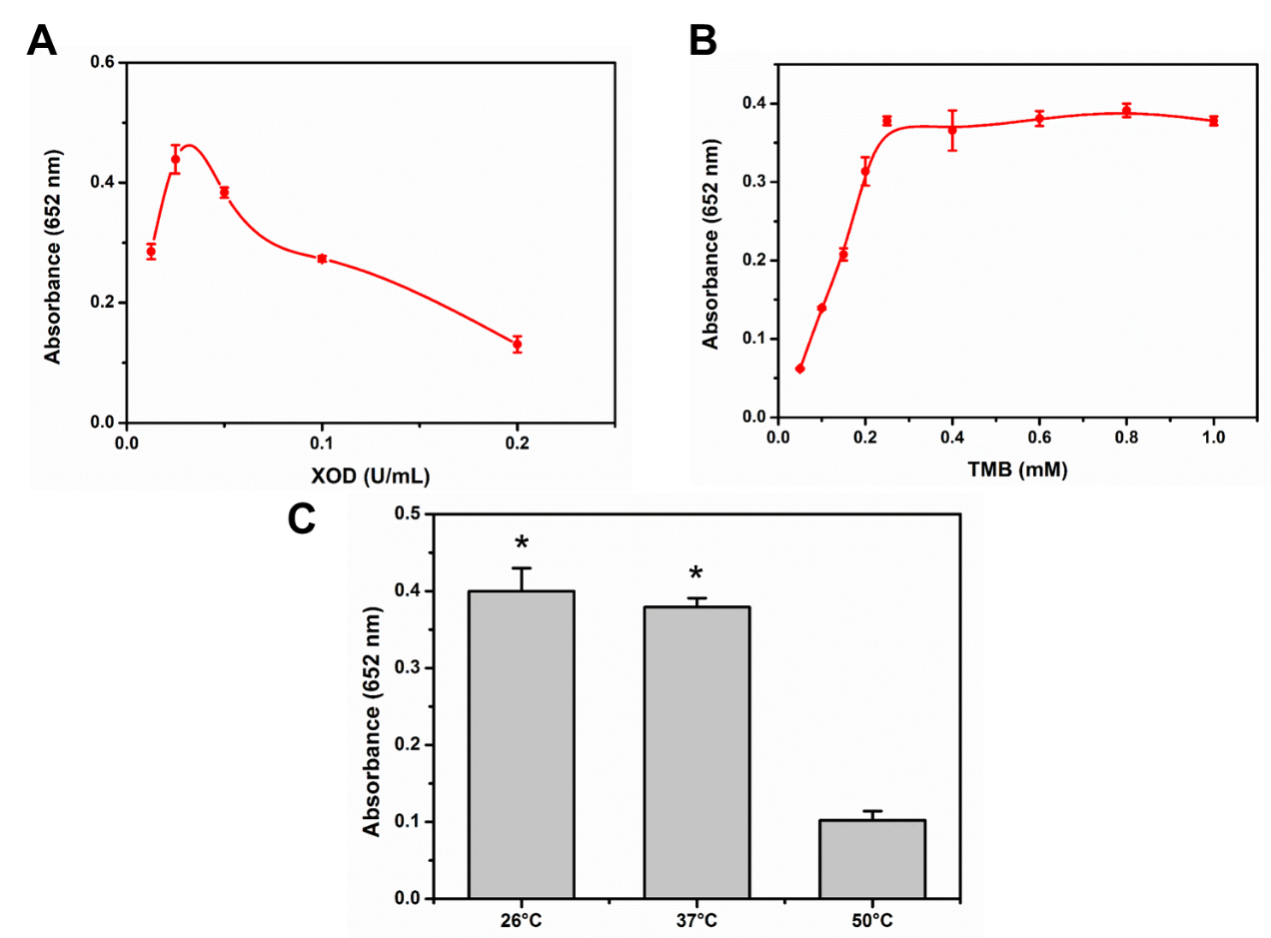
**

**Supplementary Figure 3.** Optimization of one-step hypoxanthine detection. A. Different amount of XOD. B. Varying concentration of TMB. C. Different reaction temperature. Statistical significance (p < 0.05) was shown.

**Reference**

1. Enroth, C., Eger, B. T., Okamoto, K., Nishino, T., Nishino, T., & Pai, E. F. (2000). Crystal structures of bovine milk xanthine dehydrogenase and xanthine oxidase: Structure-based mechanism of conversion. Proceedings of the National Academy of Sciences of the United States of America, 97(20), 10723–10728.

2. Atasever, A., Ozdemir, H., Gulcin, I., & Kufrevioglu, O. I. (2013). One-step purification of lactoperoxidase from bovine milk by affinity chromatography. Food Chem. 136(2), 864–870.
